# On the Extermination of the Small-Pox

**Published:** 1804-04-01

**Authors:** 


### *To the Editors of the Medical and Physical Journal.*

Gentlemen,

THE great object of all our exertions is the attainment of happiness. The prodigal and the miser, the peasant and the philosopher, all keep in view this attractive prize, but but follow it through different paths; a discovery or invention excites our approbation, as it is likely to facilitate its attainment. It is this which constitutes its excellence, and to this we must ultimately refer in examining its claim to utility. The present age teems with innovations; but amidst the varied crowd, the Cow-pox excites the greatest attention, and the most sanguine expectations. By means of its introduction, the medical profession expect to destroy a disease, which has so long continued its extensive ravages, and thus to remove a portion of evil from the world; but its future advantages remain to be questioned by philosophers and to be determined by experience. Some doubts upon the subject have lately appeared in a paper, signed Scepticus, which drew forth two answers shortly after. If I may judge from his silence, the doubts of Scepticus are perfectly removed; but as the arguments of these writers have produced no conviction in my mind, I shall proceed to the statement of my sentiments.

What are the probable consequences of the extermination of the Small-pox?

1st. I shall state the principles which are the foundation of my opinion.

2d. I shall apply them to the solution of this question.

3dly. I shall consider the arguments contained in the answers.

The human species, if supplied with ample stores of subsistence, double their numbers every twenty-five years. The United States of America bear undeniable testimony to the truth of this position. Possessed of large tracts of rich land, which was easily procured at a trifling expence, most of the settlers became cultivators for themselves. Those who had purchased larger shares than they were able to cultivate alone, found a difficulty in procuring labourers, and the price of labour rose proportionably high. A large family, far from being a burthen, was a source of opulence to the parents, and marriages were consequently early and universal. The price of provisions was extremely low, for although the labour which was employed in producing them was much dearer than in old countries, yet the rent of land which here forms so considerable part of their price was more than proportionably less. In such a state, population advanced with rapid strides, and nearly the same rapidity continues. Twenty-five years is the time in which the whole population of the States is doubled; but in some of the back settlements, where the manners are more pure and the air more salubrious, its rapidity must be greater; greater; for during a long time the number of deaths in the larger towns has exceeded the births, and a constant supply from the country was therefore necessary to support their population.

But although Nature has endued the human species with this astonishing fecundity, it is certain that their numbers must always be limitted by the means of subsistence. The food which is necessary for the maintainence of a million of inhabitants cannot afford the same subsistence to a million and half; and although the share of each individual may be somewhat lessened, there is a point at which this diminution must stop. In a country which has been inhabited for a considerable time, the only addition to the quantity of the means of subsistence must depend upon an increase of the cultivated land, or an improvement in the art of cultivation. Its increase will now be slow and gradual, and population, notwithstanding its natural rapidity, must accommodate itself to this motion. Its elastic spring will be constantly pressing against the barrier of subsistence, but the scarcity of food in a variety of forms will be as constantly acting in curtailing its exuberance. That the slow increase of the inhabitants of old countries arises from a scarcity of food is sufficiently proved by its instant rapidity on the removal of this cause. The various checks to population, which are constantly operating in this state of society, and equalizing the numbers with the means of subsistence, may be classed under two general heads.

1st. Those which diminish the number of births that would otherwise take place.

2d. Those which tend to shorten the natural term of human life.

Under the first is ranked that unwillingness to marriage, which in all modern countries has considerable influence, and which arises from the difficulty of maintaining a family. Indiscriminate intercourse of the sexes, which detroys the fruitfulness of conception. Under the second head come filth, deficiency of food, large towns, unhealthy occupations, and diseases of all kinds.

I have here endeavoured to state my principles with perspicuity and conciseness, and to be sufficiently ample for my present purpose; of their truth I have little doubt, but should any exist in the mind of the reader, I refer him to the Essay on the Principle of Population by Mr. Malthus, from which the preceding is an abstract, whose lucid reasoning will remove every obstacle to conviction, except insurmountable prejudice.

I now

I now proceed to a view of the probable consequences of the extermination of the small-pox. England is highly and extensively cultivated, and an increase of the quantity of food must be necessarily slow; here therefore a variety of checks are necessary to restrain the number of the inhabitants within the means of subsistence; and among the diseases which form so large a part, the small-pox held an eminent station. It is stated by Dr. Heberden in his Observations on the Increase and Decrease of Diseases, that of every thousand deaths the average number to be attributed to the small-pox is 95. The population of England being 9,168,000, and the annual mortality of the kingdom 1 in 40* of the inhabitants, which is 229,200, the number which annually died of this disease alone is 21,774. If so considerable a check to population be removed, no one can doubt that another equally efficacious will arise, and the only question to be asked is, whether the exchange will ultimately be beneficial? In calculations of happiness, the philosopher turns his attention to the labouring poor as the great mass of every community, and the present object of my inquiry is, in what manner they will be influenced by the removal of this check. A plenty of the necessaries of life is an essential ingredient of happiness, and the quantity in the possession of the poor depends upon the liberality with which their labour is rewarded. The wages of labour depend upon the proportion which the demand for labour bears to the quantity of labour in the market. If the demand be greater than the supply, the wages of labour will be high; but if the supply be greater than the demand, they will be proportionably low. The demand for labour depends upon the funds destined for the payment of wages, which are of two kinds: 1st. the annual revenue which is more than necessary for the maintainence; and 2d. the stock which is more than necessary for the employment of masters. It is evident that the supply of labour depends upon the number of labourers.† Since the inoculation of the small-pox has been introduced into this country, its mortality has principally fallen upon the poor, who either from carelessness, the expence of inoculation, or some other cause, have neglected to make use of that preventive, while a constant source of infection was kept up by

* Malthus's, Essay on Population.

† This subject is amply and clearly treated in Dr. A Smith's Wealth of Nations.

by it; thus many who would otherwise have lived to maintain themselves by their labour, are cut off in infancy, the supply of labourers is diminished, the wages of labour are raised, and the happiness of the poor is consequently increased. Who then can doubt the consequences of destroying this disease? A large addition will be made to the present quantity of labour, the wages will be reduced to the most miserable subsistence of the labourer, and all the evils of squalid poverty will be diffused among the most considerable class of the society. The increased price of provisions, which will be the necessary effect of the increased number of inhabitants, will lessen the demand for labourers; for in such a time of scarcity, masters will diminish the number of their servants. But though population will thus for a time be raised above its proper level, this cannot continue long. Misery, arrayed in all her terrors, will extend her destructive arm, and mankind will shrink beneath her poisonous touch. Truth obliges me to sketch this melancholy picture, and I hasten from it to a task less disagreeable.

As the paper of Credulous contained nothing of argument which militated against my opinions, that signed J. J. is the only one I have to examine; and for the sake of perspicuity, I will take the arguments in the order he has placed them.

"1. As great a number of men exist at all times as the earth is able to support." In this statement it was evidently meant by Scepticus, as many as the *present* productions of the earth are able to support, for no one can doubt that if the means of subsistence be increased, population will rise to an equal height; the answer is therefore inapplicable.

"2. Vice and misery were introduced to prevent an increase of population." There are only two ways of explaining the observations made on this statement by J. J. either he was inclined to cavilling, or he was ignorant of the nature of the question concerning the origin of evil. Philosophers have endeavoured to make the existence of evil consistent with the infinite benevolence of the Deity. A great part of the vice and misery in the world proceeds from the constant tendency of mankind to increase beyond the supply of food; this tendency is therefore in one sense its cause: but the question still presses upon the mind, why this tendency? The enquiry might be carried through a long succession of causes, up to the very plan of the creation, and the question would still demand explanation, why this plan from an infinitely wise, powerful, and benevolent Being?

"3. Disease is a species of misery, and the small-pox is a species of disease." Against this my antagonist does not contend, but he immediately adds, "the conclusion (*i. e.* the impropriety of exterminating the small-pox) cannot be allowed, for it is deduced from premises which are *doubtful* or *false*." I flatter myself I have already proved it to be deduced from premises which are *certain* and *true*.

I now come to the last observation which I have to examine: "The conclusion, if it has any weight at all, has too much; it proves the impropriety of attempting to prevent any other disease as well as the small-pox." The unavoidable necessity of checks to population being proved, the only question which remains is, which are to be preferred, as to *these* we should endeavour to transfer the influence of the others. In relation to the comparative advantages they possess, I think they may be placed in the following order.

1st. Want of inclination to marriage.

2d. Those diseases, whose mortality falls principally upon infants. The death of an infant excites a temporary uneasiness in the minds of the parents, but time soon wears away the impression, and society receives no injury.

3d. Those diseases whose mortality falls principally upon adults. The cords of affection which bind us to our worldly attachments, become more strong and numerous as age advances, and the pain from their rupture more acute; a wife and family, who depended for subsistence upon the personal exertions of the deceased, may be left destitute of support, or the burthen must fall upon society; in either cases the evil is considerable.

4th. Vice, or that indiscriminate intercourse which takes place between the sexes, destroying the fruitfulness of connection. Reason and religion oblige me to place it at the bottom of the scale.

The small-pox must certainly be placed among the second in the scale, and yet so desirable and considerable a check is in danger of being removed by the impertinent and meddling hand of man. The preceding principles incontrovertibly prove, that it is impossible to remove one check without giving rise to another; and when it amounts to so great a certainty, that the succeeding check will be a greater evil than that which we remove, the impropriety of our conduct is evident.

*March* 9, 1804. PHILANTHROPUS.

